# Crystal structure of piperidinium 4-nitro­phenolate

**DOI:** 10.1107/S1600536814025306

**Published:** 2014-11-21

**Authors:** N. Swarna Sowmya, S. Sampathkrishnan, S. Sudhahar, G. Chakkaravarthi, R. Mohan Kumar

**Affiliations:** a Department of Applied Physics, Sri Venkateswara College of Engineering, Chennai 602 117, India; b Department of Physics, Presidency College, Chennai 600 005, India; c Department of Physics, CPCL Polytechnic College, Chennai 600 068, India

**Keywords:** crystal structure, mol­ecular salt, piperidinium, 4-nitro­phenol, hydrogen bonding, C—H⋯π inter­actions

## Abstract

In the title mol­ecular salt, C_5_H_12_N^+^·C_6_H_4_NO_3_^−^, the piperidine ring adopts a chair conformation and the cation is protonated at the N atom. In the anion, the nitro group is twisted at an angle of 10.30 (11)° with respect to the attached benzene ring. In the crystal, N—H⋯O hydrogen bonds link adjacent anions and cations into infinite chains propagating along [100]. The chains are linked by C—H⋯π inter­actions, forming sheets lying parallel to (001).

## Abstract 2

In the title salt, the piperidine ring of the cation adopts a chair conformation. In the crystal, N—H⋯O hydrogen bonds link adjacent anions and cations into infinite chains along [100]. The chains are linked by C—H⋯π inter­actions, forming sheets lying parallel to (001).

## Chemical context   

Piperidine derivatives exhibit a broad-spectrum of biological activities such as anti-bacterial and anti-cancer (Parthiban *et al.*, 2005 ▶). Nitro-aromatics are widely used either as materials or as inter­mediates in explosives, dyestuffs, pesticides and organic synthesis (Yan *et al.*, 2006 ▶). We report herein on the synthesis and crystal structure of the title mol­ecular salt, prepared by mixing piperidine with 4-nitro­phenol.
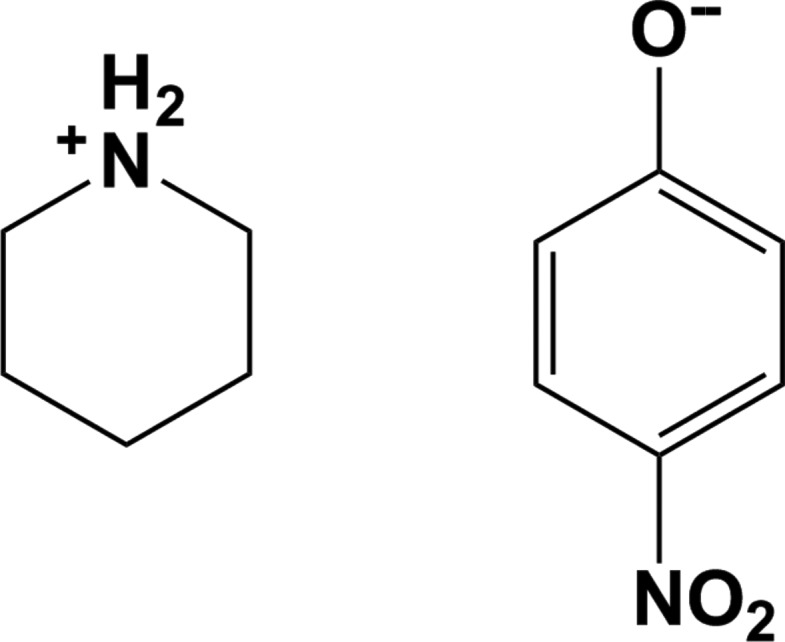


## Structural commentary   

**Figure 1:**
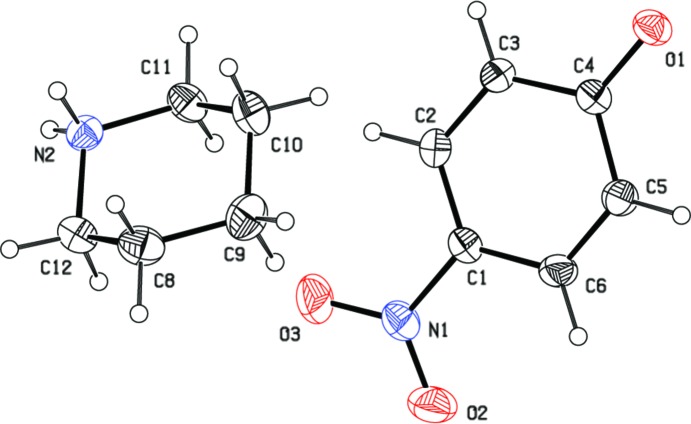
The mol­ecular structure of the title salt, showing the atom labelling. Displacement ellipsoids are drawn at the 30% probability level.

The mol­ecular structure of the title compound is illustrated in Fig. 1 ▶. The geometric parameters are close to those reported for similar structures *viz*. 1-acetyl-*c*-3,*t*-3-dimethyl-*r*-2,*c*-6-di­phenyl­piperidin-4-one (Aravindhan *et al.*, 2009 ▶), 4-nitro­phenol-piperazine (2/1) (Nagapandiselvi *et al.*, 2013 ▶) and 2-carboxyl­atopyridinium-4-nitro­phenol (1/1) (Sankar *et al.*, 2014 ▶). The piperidine ring (C8–C11/N2/C12) adopts a chair conformation with puckering parameters of *Q* = 0.5601 (17) Å, θ = 1.80 (17) and ϕ = 19 (10)°. The nitro group (N1/O2/O3) is twisted at an angle of 10.30 (11)° with respect to the attached benzene ring (C1–C6).

## Supra­molecular features   

**Figure 2:**
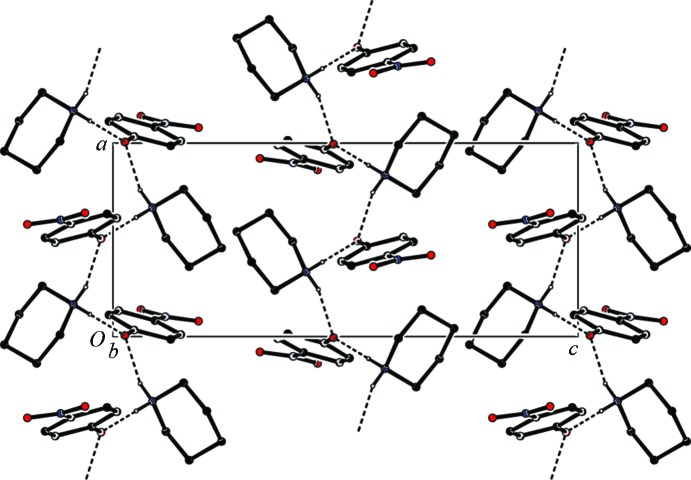
The crystal packing of the title salt, viewed along the *b* axis. Hydrogen bonds are shown as dashed lines (see Table 1 ▶ for details; H atoms not involved in hydrogen bonding have been omitted for clarity).

In the crystal, adjacent cations and anions are linked by the N—H⋯O hydrogen bonds, which generate infinite chains along [100] (see Table 1 ▶ and Fig. 2 ▶). The chains are linked by C—H⋯π inter­actions, forming sheets lying parallel to the *ab* plane (Table 1 ▶).

## Synthesis and crystallization   

Piperidine (0.85 g) and 4-nitro­phenol (1.39) in an equimolar (1:1) ratio were added to methanol as solvent and the mixture was stirred for 2 h, giving a clear solution. The solution was filtered into a beaker and sealed with parafilm and kept at room temperature for one week. Colourless crystals suitable for X-ray diffraction analysis were obtained after one week.

## Refinement   

Crystal data, data collection and structure refinement details are summarized in Table 2 ▶. The N-bound and C-bound H atoms were positioned geometrically and refined using a riding model: N—H = 0.90, C—H = 0.93 and 0.97 Å for CH and CH_2_ H atoms, respectively, and with *U*_iso_(H) = 1.2*U*_eq_(N,C).

## Supplementary Material

Crystal structure: contains datablock(s) I, global. DOI: 10.1107/S1600536814025306/su5022sup1.cif

Structure factors: contains datablock(s) I. DOI: 10.1107/S1600536814025306/su5022Isup2.hkl

Supporting information file. DOI: 10.1107/S1600536814025306/su5022Isup3.cml

CCDC reference: 1034875

Additional supporting information:  crystallographic information; 3D view; checkCIF report

## Figures and Tables

**Table 1:** Hydrogen-bond geometry (, ) *Cg*1 is the centroid of the C1C6 ring.

*D*H*A*	*D*H	H*A*	*D* *A*	*D*H*A*
N2H2*A*O1^i^	0.90	1.92	2.788(2)	161
N2H2*B*O1^ii^	0.90	1.80	2.6985(15)	175
C6H6*Cg*1^iii^	0.93	2.75	3.428(3)	130

Symmetry codes: (i) 
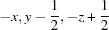
; (ii) 
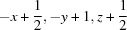
; (iii) 
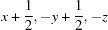
.

**Table 2:** Experimental details

Crystal data	
Chemical formula	C_5_H_12_N^+^C_6_H_4_NO_3_
*M*_r_	224.26
Crystal system, space group	Orthorhombic, *P*2_1_2_1_2_1_
Temperature (K)	295
*a*, *b*, *c* ()	6.867(5), 10.121(4), 16.497(6)
*V* (^3^)	1146.6(10)
*Z*	4
Radiation type	Mo *K*
(mm^1^)	0.10
Crystal size (mm)	0.26 0.22 0.20

Data collection	
Diffractometer	Bruker Kappa APEXII CCD
Absorption correction	Multi-scan (*SADABS*; Sheldrick, 1996 ▶)
*T*_min_, *T*_max_	0.976, 0.981
No. of measured, independent and observed [*I* > 2(*I*)] reflections	6505, 2908, 2612
*R*_int_	0.017
(sin /)_max_ (^1^)	0.673

Refinement	
*R*[*F* ^2^ > 2(*F* ^2^)], *wR*(*F* ^2^), *S*	0.035, 0.091, 1.04
No. of reflections	2908
No. of parameters	146
H-atom treatment	H-atom parameters constrained
_max_, _min_ (e ^3^)	0.13, 0.16

Computer programs: *APEX2* and *SAINT* (Bruker, 2004 ▶), *SHELXS97* and *SHELXL97* (Sheldrick, 2008 ▶) and *PLATON* (Spek, 2009 ▶).

## supplementary crystallographic information

### Crystal data

**Table:**

C_5_H_12_N^+^·C_6_H_4_NO_3_^−^	*F*(000) = 480
*M**_r_* = 224.26	*D*_x_ = 1.299 Mg m^−^^3^
Orthorhombic, *P*2_1_2_1_2_1_	Mo *K*α radiation, λ = 0.71073 Å
Hall symbol: P 2ac 2ab	Cell parameters from 658 reflections
*a* = 6.867 (5) Å	θ = 2.4–28.6°
*b* = 10.121 (4) Å	µ = 0.10 mm^−^^1^
*c* = 16.497 (6) Å	*T* = 295 K
*V* = 1146.6 (10) Å^3^	Block, colourless
*Z* = 4	0.26 × 0.22 × 0.20 mm

### Data collection

**Table:**

Bruker Kappa APEXII CCD diffractometer	2908 independent reflections
Radiation source: fine-focus sealed tube	2612 reflections with *I* > 2σ(*I*)
Graphite monochromator	*R*_int_ = 0.017
ω and φ scan	θ_max_ = 28.6°, θ_min_ = 2.4°
Absorption correction: multi-scan (*SADABS*; Sheldrick, 1996)	*h* = −6→9
*T*_min_ = 0.976, *T*_max_ = 0.981	*k* = −11→13
6505 measured reflections	*l* = −22→10

### Refinement

**Table:**

Refinement on *F*^2^	Secondary atom site location: difference Fourier map
Least-squares matrix: full	Hydrogen site location: inferred from neighbouring sites
*R*[*F*^2^ > 2σ(*F*^2^)] = 0.035	H-atom parameters constrained
*wR*(*F*^2^) = 0.091	*w* = 1/[σ^2^(*F*_o_^2^) + (0.0452*P*)^2^ + 0.1138*P*] where *P* = (*F*_o_^2^ + 2*F*_c_^2^)/3
*S* = 1.04	(Δ/σ)_max_ < 0.001
2908 reflections	Δρ_max_ = 0.13 e Å^−^^3^
146 parameters	Δρ_min_ = −0.16 e Å^−^^3^
0 restraints	Extinction correction: *SHELXL97* (Sheldrick, 2008), Fc^*^=kFc[1+0.001xFc^2^λ^3^/sin(2θ)]^-1/4^
Primary atom site location: structure-invariant direct methods	Extinction coefficient: 0.119 (6)

### Special details

**Table:**

Geometry. All esds (except the esd in the dihedral angle between two l.s. planes) are estimated using the full covariance matrix. The cell esds are taken into account individually in the estimation of esds in distances, angles and torsion angles; correlations between esds in cell parameters are only used when they are defined by crystal symmetry. An approximate (isotropic) treatment of cell esds is used for estimating esds involving l.s. planes.
Refinement. Refinement of F^2^ against ALL reflections. The weighted R-factor wR and goodness of fit S are based on F^2^, conventional R-factors R are based on F, with F set to zero for negative F^2^. The threshold expression of F^2^ > 2sigma(F^2^) is used only for calculating R-factors(gt) etc. and is not relevant to the choice of reflections for refinement. R-factors based on F^2^ are statistically about twice as large as those based on F, and R- factors based on ALL data will be even larger.

### Fractional atomic coordinates and isotropic or equivalent isotropic displacement parameters (Å^2^)

**Table:**

	*x*	*y*	*z*	*U*_iso_*/*U*_eq_	
C1	0.08265 (18)	0.22326 (12)	0.09031 (7)	0.0380 (3)	
C2	0.0102 (2)	0.31257 (12)	0.14583 (7)	0.0404 (3)	
H2	−0.0220	0.2848	0.1979	0.049*	
C3	−0.0144 (2)	0.44223 (12)	0.12419 (7)	0.0403 (3)	
H3	−0.0638	0.5014	0.1621	0.048*	
C4	0.03355 (18)	0.48874 (11)	0.04556 (7)	0.0373 (3)	
C5	0.1115 (2)	0.39408 (13)	−0.00839 (8)	0.0474 (3)	
H5	0.1491	0.4211	−0.0600	0.057*	
C6	0.1336 (2)	0.26352 (13)	0.01258 (8)	0.0465 (3)	
H6	0.1820	0.2029	−0.0247	0.056*	
C8	0.6387 (2)	0.21154 (17)	0.33603 (10)	0.0570 (4)	
H8A	0.7057	0.2749	0.3702	0.068*	
H8B	0.7348	0.1503	0.3149	0.068*	
C9	0.5422 (3)	0.28329 (19)	0.26652 (10)	0.0626 (4)	
H9A	0.6386	0.3342	0.2371	0.075*	
H9B	0.4854	0.2197	0.2294	0.075*	
C10	0.3854 (3)	0.37423 (16)	0.29817 (10)	0.0545 (4)	
H10A	0.4442	0.4428	0.3312	0.065*	
H10B	0.3199	0.4164	0.2530	0.065*	
C11	0.2391 (2)	0.29893 (14)	0.34801 (9)	0.0480 (3)	
H11A	0.1444	0.3601	0.3703	0.058*	
H11B	0.1703	0.2370	0.3135	0.058*	
C12	0.4907 (2)	0.13639 (13)	0.38617 (9)	0.0488 (3)	
H12A	0.4341	0.0663	0.3537	0.059*	
H12B	0.5549	0.0963	0.4324	0.059*	
N1	0.10251 (18)	0.08685 (11)	0.11247 (8)	0.0487 (3)	
N2	0.33415 (17)	0.22610 (10)	0.41493 (6)	0.0405 (3)	
H2A	0.2438	0.1784	0.4415	0.049*	
H2B	0.3851	0.2845	0.4502	0.049*	
O1	0.00554 (15)	0.61052 (8)	0.02564 (6)	0.0458 (2)	
O2	0.1387 (2)	0.00445 (11)	0.06015 (8)	0.0730 (4)	
O3	0.0795 (2)	0.05548 (12)	0.18390 (7)	0.0771 (4)	

### Atomic displacement parameters (Å^2^)

**Table:**

	*U*^11^	*U*^22^	*U*^33^	*U*^12^	*U*^13^	*U*^23^
C1	0.0355 (6)	0.0359 (5)	0.0427 (6)	0.0007 (5)	−0.0004 (5)	0.0057 (5)
C2	0.0423 (6)	0.0453 (6)	0.0337 (5)	−0.0013 (6)	−0.0019 (5)	0.0049 (5)
C3	0.0434 (7)	0.0414 (6)	0.0361 (6)	0.0028 (5)	0.0005 (5)	−0.0033 (5)
C4	0.0324 (6)	0.0364 (6)	0.0431 (6)	−0.0006 (5)	0.0018 (5)	0.0030 (5)
C5	0.0545 (8)	0.0445 (7)	0.0432 (7)	0.0065 (6)	0.0179 (6)	0.0089 (5)
C6	0.0517 (8)	0.0412 (6)	0.0466 (7)	0.0096 (6)	0.0155 (6)	0.0019 (5)
C8	0.0425 (7)	0.0574 (8)	0.0710 (10)	0.0055 (7)	0.0071 (7)	−0.0019 (7)
C9	0.0635 (10)	0.0723 (10)	0.0520 (8)	−0.0044 (9)	0.0162 (8)	0.0073 (8)
C10	0.0568 (9)	0.0489 (8)	0.0577 (8)	−0.0017 (7)	−0.0023 (7)	0.0146 (7)
C11	0.0388 (7)	0.0459 (7)	0.0594 (8)	0.0031 (6)	−0.0029 (6)	0.0053 (6)
C12	0.0495 (8)	0.0408 (6)	0.0559 (7)	0.0053 (6)	−0.0015 (7)	0.0016 (6)
N1	0.0516 (7)	0.0396 (6)	0.0547 (7)	−0.0015 (5)	0.0013 (6)	0.0090 (5)
N2	0.0433 (6)	0.0377 (5)	0.0406 (5)	−0.0053 (4)	0.0014 (4)	−0.0029 (4)
O1	0.0494 (5)	0.0346 (4)	0.0535 (5)	0.0023 (4)	0.0079 (5)	0.0061 (4)
O2	0.1004 (10)	0.0403 (5)	0.0782 (8)	0.0118 (6)	0.0251 (7)	0.0021 (5)
O3	0.1256 (13)	0.0504 (6)	0.0554 (6)	−0.0028 (7)	−0.0012 (7)	0.0188 (5)

### Geometric parameters (Å, º)

**Table:**

C1—C2	1.3798 (18)	C9—C10	1.510 (2)
C1—C6	1.3902 (18)	C9—H9A	0.9700
C1—N1	1.4347 (17)	C9—H9B	0.9700
C2—C3	1.3703 (18)	C10—C11	1.506 (2)
C2—H2	0.9300	C10—H10A	0.9700
C3—C4	1.4187 (17)	C10—H10B	0.9700
C3—H3	0.9300	C11—N2	1.4793 (18)
C4—O1	1.2900 (15)	C11—H11A	0.9700
C4—C5	1.4129 (18)	C11—H11B	0.9700
C5—C6	1.374 (2)	C12—N2	1.4848 (19)
C5—H5	0.9300	C12—H12A	0.9700
C6—H6	0.9300	C12—H12B	0.9700
C8—C9	1.511 (2)	N1—O2	1.2257 (17)
C8—C12	1.516 (2)	N1—O3	1.2306 (16)
C8—H8A	0.9700	N2—H2A	0.9000
C8—H8B	0.9700	N2—H2B	0.9000
			
C2—C1—C6	120.73 (12)	H9A—C9—H9B	108.2
C2—C1—N1	119.70 (11)	C11—C10—C9	110.87 (13)
C6—C1—N1	119.55 (12)	C11—C10—H10A	109.5
C3—C2—C1	119.92 (11)	C9—C10—H10A	109.5
C3—C2—H2	120.0	C11—C10—H10B	109.5
C1—C2—H2	120.0	C9—C10—H10B	109.5
C2—C3—C4	121.83 (12)	H10A—C10—H10B	108.1
C2—C3—H3	119.1	N2—C11—C10	111.42 (13)
C4—C3—H3	119.1	N2—C11—H11A	109.3
O1—C4—C5	122.95 (11)	C10—C11—H11A	109.3
O1—C4—C3	121.02 (11)	N2—C11—H11B	109.3
C5—C4—C3	116.03 (11)	C10—C11—H11B	109.3
C6—C5—C4	122.36 (12)	H11A—C11—H11B	108.0
C6—C5—H5	118.8	N2—C12—C8	110.68 (12)
C4—C5—H5	118.8	N2—C12—H12A	109.5
C5—C6—C1	119.09 (12)	C8—C12—H12A	109.5
C5—C6—H6	120.5	N2—C12—H12B	109.5
C1—C6—H6	120.5	C8—C12—H12B	109.5
C9—C8—C12	111.16 (14)	H12A—C12—H12B	108.1
C9—C8—H8A	109.4	O2—N1—O3	121.66 (13)
C12—C8—H8A	109.4	O2—N1—C1	119.64 (12)
C9—C8—H8B	109.4	O3—N1—C1	118.70 (12)
C12—C8—H8B	109.4	C11—N2—C12	112.69 (11)
H8A—C8—H8B	108.0	C11—N2—H2A	109.1
C10—C9—C8	110.09 (13)	C12—N2—H2A	109.1
C10—C9—H9A	109.6	C11—N2—H2B	109.1
C8—C9—H9A	109.6	C12—N2—H2B	109.1
C10—C9—H9B	109.6	H2A—N2—H2B	107.8
C8—C9—H9B	109.6		
			
C6—C1—C2—C3	0.8 (2)	C12—C8—C9—C10	56.35 (18)
N1—C1—C2—C3	−178.12 (12)	C8—C9—C10—C11	−56.27 (19)
C1—C2—C3—C4	−0.2 (2)	C9—C10—C11—N2	55.67 (18)
C2—C3—C4—O1	178.45 (13)	C9—C8—C12—N2	−55.31 (17)
C2—C3—C4—C5	−1.21 (19)	C2—C1—N1—O2	169.09 (14)
O1—C4—C5—C6	−177.44 (14)	C6—C1—N1—O2	−9.8 (2)
C3—C4—C5—C6	2.2 (2)	C2—C1—N1—O3	−9.8 (2)
C4—C5—C6—C1	−1.8 (2)	C6—C1—N1—O3	171.33 (14)
C2—C1—C6—C5	0.2 (2)	C10—C11—N2—C12	−55.30 (16)
N1—C1—C6—C5	179.09 (14)	C8—C12—N2—C11	54.81 (16)

### Hydrogen-bond geometry (Å, º)

Cg1 is the centroid of the C1–C6 ring.

**Table:**

*D*—H···*A*	*D*—H	H···*A*	*D*···*A*	*D*—H···*A*
N2—H2*A*···O1^i^	0.90	1.92	2.788 (2)	161
N2—H2*B*···O1^ii^	0.90	1.80	2.6985 (15)	175
C6—H6···*Cg*1^iii^	0.93	2.75	3.428 (3)	130

Symmetry codes: (i) −*x*, *y*−1/2, −*z*+1/2; (ii) −*x*+1/2, −*y*+1, *z*+1/2; (iii) *x*+1/2, −*y*+1/2, −*z*.
